# Climbing the ladder: a ranking approach to burnout prediction

**DOI:** 10.3389/fdgth.2025.1694666

**Published:** 2026-01-02

**Authors:** Alvise Dei Rossi, Davide Marzorati, Radoslava Švihrová, Jürg Grossenbacher, Vladislav Kochergin, Max Grossenbacher, Francesca Faraci

**Affiliations:** 1 Department of Innovative Technologies, Institute of Digital Technologies for Personalized Healthcare, University of Applied Sciences and Arts of Southern Switzerland, Lugano, Switzerland; 2 Faculty of Informatics, Università della Svizzera italiana, Lugano, Switzerland; 3 Institute of Computer Science, Faculty of Science, University of Bern, Bern, Switzerland; 4 Resilient SA, Lausanne, Switzerland; 5 Psy Bern AG, Bern, Switzerland

**Keywords:** burnout, machine learning, ranking, siamese architecture, wearable devices

## Abstract

Validated psychological assessment tools, such as the Shirom–Melamed Burnout Measure (SMBM), are essential for reliably assessing burnout. However, their reliance on active, self-reported input limits their suitability for continuous monitoring and early detection, and introduces the potential for human bias. The SMBM specifically targets the energy depletion component of burnout, with items organized into three subscales: Physical Fatigue (PF), Cognitive Weariness (CW), and Emotional Exhaustion (EE). In the present work, we investigate the feasibility of predicting burnout risk unobtrusively using the preceding trajectory of passive physiological data from wearable devices, supplemented by baseline demographic and occupational information. We evaluate classification, regression, and learning-to-rank formulations for the prediction of SMBM subscale scores on a 9-month real-world dataset of 239 workers, using both aggregate-based and sequential models. Binary classification yields modest performance [ROC AUC: PF (0.66), CW (0.67), EE(0.56)], and regression models offer negligible gains over naïve benchmarks. However, rank-based metrics suggest relative burnout severity can be partially inferred from wearable signals. Motivated by this, we propose a siamese recurrent neural network, explicitly tailored for sequential wearable data and optimized for pairwise risk estimation. Results show improved alignment with the ordinal nature of burnout scores for PF (Spearman’s ρ=0.29, Normalized Discounted Cumulative Gain =0.93) and CW (Spearman’s ρ=0.25, Normalized Discounted Cumulative Gain = 0.89), whereas assessing EE may require additional modalities. Although real-world implementation remains challenging, ranking-based techniques could pave the way for more effective burnout risk monitoring.

## 1 Introduction

Burnout is classified in the ICD-11 as an occupational phenomenon resulting from chronic workplace stress that has not been successfully managed, characterized by energy depletion, increased cynicism toward one’s job, and reduced professional efficacy [1, 2]. Burnout is widespread across diverse professions, especially among healthcare workers [3–5], with rates rising sharply during the COVID-19 pandemic [6], and shows distinct patterns between white- and blue-collar workers [7], heightened vulnerability in shift workers [8], while also affecting broader segments of the population beyond the workplace [9]. Elevated burnout levels are frequently associated with somatic complaints such as chronic fatigue, gastrointestinal disturbances, and sleep disruptions [9], and have been linked to increased risk of type 2 diabetes [10], cardiovascular risk [11], and reduced vagal tone, a marker of impaired parasympathetic regulation [12]. Furthermore, burnout is closely tied to depression and anxiety, with meta-analytic evidence revealing substantial correlations (r=0.52 and r=0.46, respectively) [13], though the constructs are not isomorphic [13, 14]. Beyond individual health consequences, burnout also imposes significant economic costs on organizations and society at large [15, 16]. Given its high prevalence and broad impact, burnout requires urgent attention through effective prevention and early detection strategies, crucial for enabling timely intervention.

Effective prevention efforts rely on the accurate assessment of burnout. Validated self-report tools include the widely used Maslach Burnout Inventory (MBI), which evaluates emotional exhaustion, depersonalization, and reduced personal accomplishment [17]; the Shirom–Melamed Burnout Measure (SMBM), which captures burnout across cognitive weariness, emotional exhaustion, and physical fatigue [18, 19]; and the Copenhagen Burnout Inventory (CBI), which emphasizes fatigue and exhaustion as central symptoms [20]. While standardized self-report instruments remain the cornerstone for burnout assessment, they require individuals to actively initiate evaluation, consulting a healthcare professional, filling in questionnaires, and participating in interviews. Because these methods rely on individuals’ willingness and initiative to report their feelings and undergo assessment, detection may be delayed, particularly in the early stages of burnout when signs may be subtle. In this context, wearable devices present a promising, though still emerging, avenue for early identification and continuous monitoring of burnout risk [21, 22]. These devices, ranging from wristbands to smart rings, enable the unobtrusive collection of physiological and behavioral data, including cardiac metrics, sleep characteristics, and physical activity patterns [23, 24]. Growing evidence indicates that these signals are associated with mental health conditions and hold potential to support clinical decision-making in these areas [25]. In occupational contexts, research suggests that physiological indicators gathered through wearables may contribute to enhanced work performance and support the well-being of employees [21], although current implementations are still largely constrained [26].

Recent research has increasingly examined physiological markers of burnout that can be tracked through wearable devices. Sleep disruption is among the most consistent findings: individuals with burnout show fragmented sleep, reduced slow-wave and REM stages, lower sleep efficiency, and persistent daytime fatigue [27, 28], while prospective studies identify poor sleep quality and short duration as key contributors to burnout onset [29]. Physical activity has also been associated with burnout, with evidence suggesting that regular movement, both during work-time and leisure-time, may lower risk [30, 31]. Additionally, burnout has been associated with increased heart rate and reduced heart rate variability (HRV), reflecting heightened physiological stress responses and lower parasympathetic activity [12, 32, 33]. While wearable sensing in naturalistic settings faces inherent limitations in capturing correlates of stress in real time without contextual information [34, 35], recent deep learning methods that integrate time- and frequency-domain features across multiple physiological modalities have shown promising performance in detecting short-term stress in certain occupational contexts [36], though further validation to larger cohorts and unseen subjects are warranted. Moreover, we note that substantially higher accuracy in the measurement of HRV can be achieved when considering resting conditions, i.e., overnight measurements [37]. Beyond physiological indicators, contextual and demographic factors also contribute meaningfully to burnout risk. The causal progression of burnout symptoms may differ between men and women [38], with several studies identifying gender as a significant predictor [9, 19, 39].

Resilience refers to an individual’s ability to maintain or regain mental well-being in the face of adversity. It has been shown to reduce the severity of burnout by protecting against emotional exhaustion and enhancing feelings of personal accomplishment [40, 41]. Building on this link, recent studies have begun modeling the dynamic relationship between resilience and mental health. Adler et al. [42] proposed a framework combining wearable sensors with ecological momentary assessments to detect markers of resilience, operationalized as individuals exhibiting minimal change in the patient health questionnaire-9 (PHQ-9) scores over time. Their density-estimation approach identified step count and sleep duration as key mobile-sensing features in high-stress contexts. More recently, large-scale longitudinal paradigms have sought to capture resilience processes over time by repeatedly monitoring exposure to both major life events and daily microstressors, alongside fluctuations in mental health [43]. Kalisch et al. [44] introduced a normative modeling approach inferring resilience from an individual’s residuals from the expected relationship between stressor exposure and mental health symptoms. However, these designs rely heavily on frequent self-reporting, and replacing these with objective features may offer a less burdensome alternative [44]. An early attempt by [45] used the same resilience definition of [44] in a machine learning framework to predict individual resilience scores from both daily-life psychological and physiological data. While psychological-based models performed moderately well, physiological-based models underperformed. Overall, whether predicting burnout directly or via resilience-based proxies, models based solely on physiological data continue to face significant challenges. Most studies report limited or negative results [26], with only a few achieving above-chance performance [42, 46], highlighting the need for further methodological advancement.

In this work, we explore fully unobtrusive [47] burnout prediction by estimating burnout risk using only passively collected data, without requiring active user input. We predict burnout scores based on prior trajectories of daily physiological markers, considering both aggregated features and sequential architectures, enriched with demographic and occupational data collected at baseline, assuming no access to prior burnout assessments. We begin by outlining the performance and limitations of conventional classification and regression approaches. Then, drawing from the observation that both burnout and resilience are often defined in relative terms, either in terms of deviation from the norm [43–45], from stress-resilient outcomes [42], or via population percentiles [48], we propose a shift toward a ranking-based formulation of burnout risk prediction. To the best of our knowledge, this is the first study to explicitly frame the task within a learning-to-rank paradigm [49]. Models are trained to learn the relative severity of burnout across individuals, focusing on the correct ordering rather than predicting absolute values or categories. To implement this, we propose a siamese recurrent neural network [50], drawing inspiration from the RankNet approach [51].

## 2 Materials and methods

### 2.1 The dataset

The data analyzed in this study were collected as part of the “Risk Identification and Prevention of Work-Related Stress Disorders” (WRSD) project. The study design adhered to the Declaration of Helsinki guidelines [52] (protocol number 628-2023-SPER-AUSLBO, Comitato Etico Area Vasta Emilia Centro, Italy; Clarification of Responsibility Req-2023-00377, SwissEthics, Switzerland). The detailed methodology, participant characteristics, and adherence analysis of the WRSD protocol are thoroughly described in another paper by the authors [53]. Here, we summarize all the relevant characteristics of the original dataset and the data collection protocol for the present work. The cohort considered comprised N=239 office and production workers from Italy and Switzerland, monitored over a 9-month period (mean ± SD age 38±8 years; 40% female). Each participant was provided with a Garmin Venu Sq 2 smartwatch and a custom mobile application, enabling continuous monitoring of physiological parameters. The devices captured minute-level heart rate, 15-min activity epochs, 3-min stress and breathing rate, and detailed sleep metrics, including sleep composition and nightly summaries. Burnout assessments were administered monthly within the application using the 14-item version of the Shirom-Melamed Burnout Measure [10], evaluating physical fatigue (PF), cognitive weariness (CW), and emotional exhaustion (EE) components. The survey consisted of 14 questions asking participants to report how often, over the past 30 days, they experienced specific feelings, using a 7-point Likert scale ranging from 1 (“*Never or almost never*”) to 7 (“*Always or almost always*”); the resulting component scores and the overall score also ranged from 1 to 7. At baseline, alongside their initial baseline SMBM assessment, participants also completed socio-demographic and work-related questionnaires.

### 2.2 Window extraction and feature representation

Since each SMBM survey prompts participants to reflect on their experiences over the preceding 30 days, in this work we base our predictions on physiological data collected from the 20- to 30-day window preceding each assessment. Shorter windows are used when the previous SMBM submission occurred less than 30 days earlier, ensuring no temporal overlap. Baseline assessments are excluded, as they lack prior physiological data and were modeled separately [46]. Each window is required to include at least 10 adherent days and 10 adherent nights. This requirement is guided by recent literature, showing that accurate estimates of monthly sleep metrics could be obtained from 10 daily observations [54]. We define adherent days as those with ≥70% of device wearing time, while adherent nights as nights with non-manually reported sleep summaries from the device, restricted to primary sleep sessions lasting at least two hours. The window extraction process is illustrated in Supplementary Figure S1.

Given the relatively limited amount of labeled data, we restrict our modeling to a focused subset of time-varying wearable-derived features that have demonstrated associations with burnout in prior research [27–29, 31, 32]. Within the sleep domain, we include total sleep time, time spent awake after sleep onset, and restorative sleep percentage, defined as the combined proportion of deep and REM sleep. For cardiac parameters, we select a daily and a nocturnal feature: median heart rate while awake and rest heart rate, i.e., the lowest 30-min moving average while sleeping. Since direct HRV measures aren’t available, we incorporate the mean overnight Garmin stress score as a proxy [55]. To summarize daily activity patterns, we consider the percentage of time spent in a sedentary state. We also incorporate a subset of time-invariant features from the onboarding socio-demographic and occupational assessments: gender [38], age, BMI, relationship status (defined as being in a stable relationship or not), and binary occupational variables such as seniority, work type (production/office), and engagement in shift work [8]. For time-varying features and SMBM assessments we assess the intra-class correlation coefficient (ICC) [56], quantifying the proportion of variance attributable to between- vs. within-person differences, with values closer to 1 indicating greater between-person variability.

### 2.3 Burnout prediction

All models are trained and evaluated using stratified (nested) 10-fold cross-validation, with subject-wise splits [57], and stratification achieved by binning the target scores into quartiles. Throughout all experiments, SMBM components (PF, CW, and EE) are treated as distinct outcomes and predicted independently.

**Figure 1:**
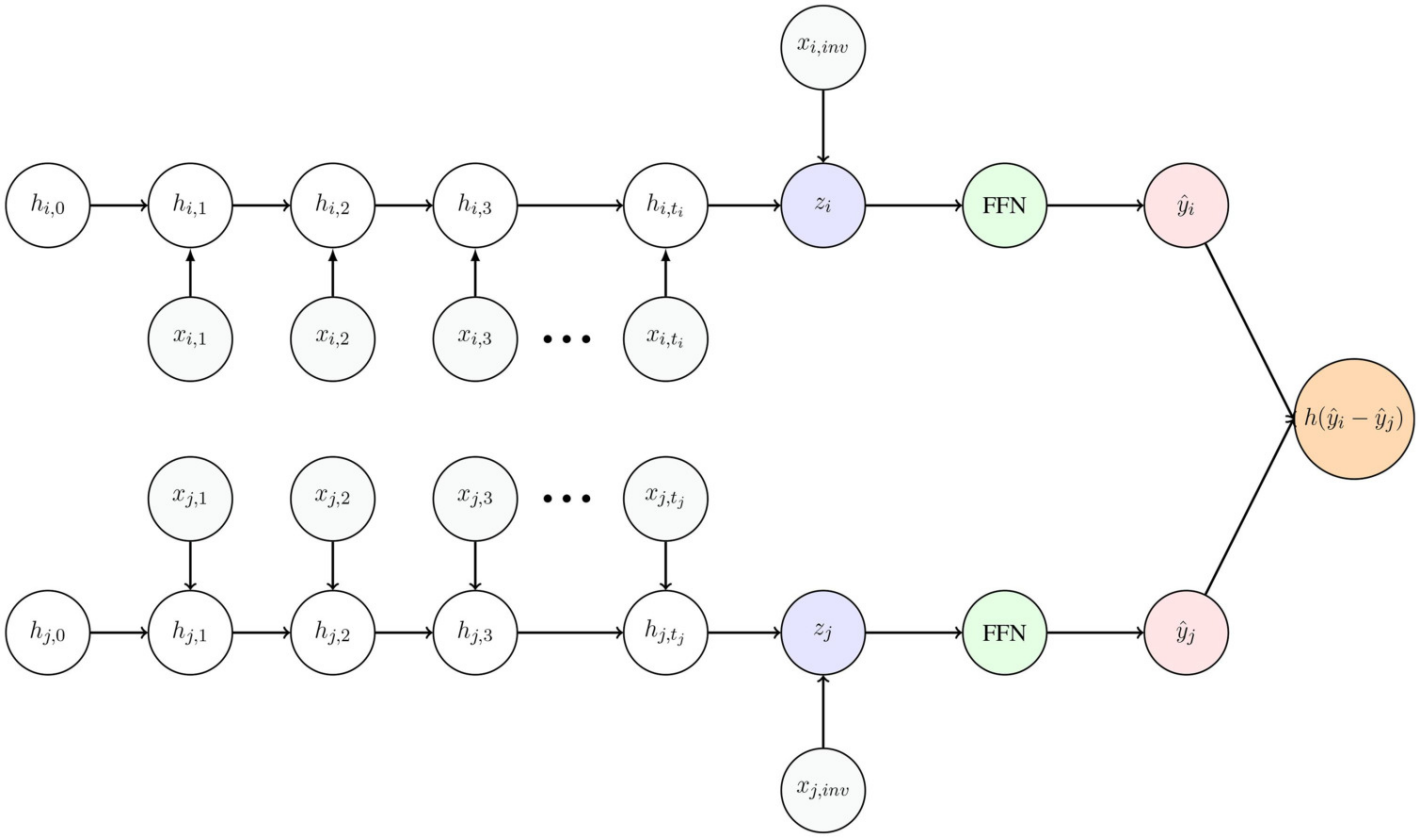
Pairwise ranking architecture for sequences i and j. Each sequence is processed by a recurrent encoder with shared weights. The encoder takes as input the time-dependent features $x_{i,t}$, $x_{\;j,t}$ and computes a series of hidden states $h_{i,t}$, $h_{\;j,t}$. The final hidden states $h_{i,t_{i}}$, $h_{\;j,t_{j}}$ are concatenated with the corresponding time-invariant features $x_{i,\text{inv}}$, $x_{\;j,\text{inv}}$, and passed through fully connected layers to produce scalar scores ${\hat{y}}_{i}$ and ${\hat{y}}_{j}$. These scores are then compared using a comparison function $h({\hat{y}}_{i},{\hat{y}}_{j})$ to learn the relative ordering between the two instances.

We consider two modeling approaches to predict burnout from physiological data. The first relies on aggregating features across the extracted time windows, similarly to what was done in [45, 46]. For each time-varying feature, we compute a set of summary statistics, including the mean, standard deviation, 25th and 75th percentiles, skewness, kurtosis, and the number of zero-crossings. These aggregated features, combined with time-invariant variables, are used as inputs to traditional machine learning models. To mitigate dimensionality issues, recursive feature selection [58] is performed to identify a subset of 15 features. As a second approach, we model the temporal dynamics of physiological features within each window using Gated Recurrent Unit (GRU) recurrent neural networks (RNNs) [59]. Daily observations serve as time steps, processing sequences of varying lengths by padding shorter windows and ignoring the padded time steps. Each sequence of daily observations is encoded into a fixed-length representation, which is concatenated with time-invariant features. This joint representation is passed through a feedforward neural network (FFN) with a single hidden layer and dropout to produce the final scalar output. The network described is represented in the upper branch of Figure 1. Training is performed using the Adam optimizer ($\eta =10^{-5}$) [60] for up to 100 epochs, with early stopping based on validation loss and a patience of 10 epochs.

We first evaluate both modeling strategies within standard classification and regression frameworks. For classification, burnout scores are dichotomized into “low” and “high” risk categories according to SMBM cut-off values (CW: 2.83, EE: 2.75, PF: 3.5) provided in [48]. In regression, the models are tasked with predicting the continuous SMBM component scores directly. We evaluate a different range of machine learning algorithms: for classification, we employ Logistic regression, Linear and Quadratic Discriminant Analysis, and Random Forest; for regression, we use Lasso and Ridge regressors, Elastic Net, and Random Forest. For sequential modeling, the architecture is held constant and only the loss function is adapted to the task. Classification models are trained to minimize binary cross-entropy and are evaluated using ROC-AUC and F1 score of the high-risk class. Regression models are trained to minimize mean squared error and are evaluated using mean absolute error (MAE) and Spearman’s ρ [61]. The hyperparameter space explored for each model can be found in Supplementary Table S2.

We then frame burnout prediction as a ranking task. Learning to rank is a supervised machine learning framework that has evolved primarily within information retrieval systems but represents a general paradigm for problems where the primary objective is the accurate ordering of instances rather than absolute prediction [49, 62]. In ranking frameworks, training data consist of sets of items with partial ordering relationships specified between items, enabling models to learn relative preferences. In our context, ranking is expressed as a single query task, as it seeks to determine the relative burnout risk ordering among individuals within the given population. Unlike classification approaches, ranking eliminates the need to select potentially arbitrary thresholds for distinguishing between risk categories. Moreover, regression can be seen as the most basic form of ranking, i.e., point-wise ranking, as it treats each instance independently, ignoring the relational structure inherent in comparative assessments. Instead, in a pairwise ranking approach, models are trained on pairs of instances with the objective of learning their relative order. This setup is commonly framed as a binary classification task: for each pair $\left(x_{i},x_{j}\right)$, the model predicts whether $x_{i}$ should be ranked higher than $x_{j}$. This is typically achieved by defining a shared scoring function f that maps each instance x to a real-valued score, and then comparing the scores $f(x_{i})$ and $f(x_{j})$ with a function $h(f(x_{i}),f(x_{j}))$. RankNet [51] is a seminal pairwise algorithm that formalizes this process: a feed-forward network computes the scores $f(x_{i})$ and $f(x_{j})$ and their difference is passed through a sigmoid to yield the probability that $x_{i}$ should be ranked above $x_{j}$, with training based on binary cross-entropy loss (BCE). Building on the RankNet framework, we employ a siamese recurrent neural network [50, 59] specifically designed for ranking longitudinal data. Each branch of the siamese network, with weights shared across the branches, mirrors the GRU-based sequential model described earlier. Given two instances i and j, the network computes scores ${\hat{y}}_{i}$ and ${\hat{y}}_{j}$, which are compared to infer their relative ordering. We report an intuitive schematic of the proposed architecture in Figure 1. This approach allows to leverage the strengths of pairwise ranking, namely avoidance of thresholds and the focus on relative ordering, while modeling temporal patterns in the data. We restrict training to pairs of windows where the absolute difference in the burnout scores is at least 0.5, acknowledging that small differences in self-reported scores may not be significant. We experiment with two different loss functions: BCE, as used in RankNet [51], and the Margin Ranking Loss (MRL), a margin-based alternative commonly used in learning-to-rank tasks:

$$L_{\text{MRL}}=\max(0,-y_{ij}\cdot ({\hat{y}}_{i}-{\hat{y}}_{j})+\epsilon ),$$

where $y_{ij}\in \{-1,1\}$ denotes the ground-truth ordering: $y_{ij}=1$ if instance i should be ranked above j, and $y_{ij}=-1$ otherwise. The margin hyperparameter ϵ>0, set to 0.5 in our experiments, defines the desired separation between the scores of correctly ranked pairs.

A detailed description of the training procedure for the siamese ranking approach is reported in Supplementary Algorithm 1.

To assess the effectiveness of ranking models, we employ Spearman correlation [61] and the Normalized Discounted Cumulative Gain (NDCG) [63]. NDCG [63] is a widely used position-sensitive metric in information retrieval that evaluates ranked lists by rewarding highly relevant items appearing earlier, with their contribution discounted logarithmically based on position. Formally:

$$\text{DCG}=\sum_{i=1}^{n}\frac{2^{rel_{i}}-1}{\log_{2}(i+1)};\;\text{NDCG}=\frac{\text{DCG}}{\text{IDCG}}$$

where $rel_{i}$ is the ground-truth relevance score of the item at rank i, and IDCG is the DCG of the ideal ranking.

## 3 Results

After excluding individuals with minimal data contribution (<14 cumulative days), 206 participants were retained for analysis. These participants provided a total of 579 valid SMBM responses, representing 28% of the expected entries. Following the exclusion of baseline assessments and the application of the adherence criteria detailed in Section 2, a final set of 258 valid windows from 129 participants was selected for modeling. Of these, 237 spanned the full 30-day period, while the remainder ranged from 20 to 29 days. Average night and daily adherence were 82.7% and 66.4%, respectively. Missing values in time-varying features were imputed using the within-window median, while feature aggregates were computed exclusively from non-imputed data. Supplementary Table S1 presents the ICC values for physiological features and SMBM assessments. SMBM scores displayed high between-person variance, with ICCs ranging from 0.65 to 0.71, supporting its temporal stability [64]. Physiological features related to sleep, activity, and stress showed lower ICCs, reflecting greater within-person variability, while cardiac features showed higher between-person variability. Burnout risk scores were derived from participants’ SMBM responses by computing individual scores for each SMBM subscale and calculating the overall burnout score as their weighted average [10, 18]. Across the analyzed windows, the mean ± SD scores were 3.56±1.28 for PF, 2.98±1.30 for CW, 2.59±1.29 for EE, and 3.04±1.09 for the overall burnout score. Modeling the overall burnout score directly, either as a composite metric or via joint modeling of the three subscales, consistently underperformed compared to modeling each component independently. Accordingly, we focus on results from separate subscale modeling. All statistical tests in the following sub-sections refer to one-sided Wilcoxon signed-rank tests, with a significance level α=0.05.

### 3.1 Classification

Table 1 presents the ROC-AUC and F1-scores for the high-risk class across ten outer folds of nested cross-validation. As a baseline, we include a biased random guesser (BRG) that samples outcomes based on the empirical label distribution. The best performances obtained for the PF and CW components are comparable, significantly surpassing the baseline, with the GRU model achieving the highest scores by a small margin. Median ROC-AUCs for GRU are 0.66 (PF) and 0.67 (CW), with corresponding median F1-scores of 0.67 and 0.62. For EE, all models perform worse; logistic regression achieves the highest ROC-AUC (0.56), but does not significantly outperform BRG, and F1-scores remain uniformly low.

**Table 1:** Median (p25–p75) ROC-AUC and F1-scores for binary classification of burnout risk across the three SMBM components.

Model	PF		CW		EE	
	ROC-AUC	F1	ROC-AUC	F1	ROC-AUC	F1
BRG	0.50(0.49,0.53)	0.57(0.55,0.57)	0.50(0.50,0.51)	0.48(0.45,0.51)	0.48(0.47,0.50)	0.35(0.33,0.39)
LR	0.59(0.48,0.67)	0.59(0.47,0.69)	0.57(0.43,0.62)	0.47(0.39,0.62)	0.56(0.53,0.69)	0.06(0.00,0.26)
LDA	${0.66(0.48,0.72)}^{\text{‡}}$	$0.66(0.50,0.71{)}^{\text{‡}}$	${0.67(0.57,0.72)}^{\text{‡}}$	0.50(0.37,0.67)	0.52(0.45,0.58)	0.27(0.23,0.33)
QDA	${0.66(0.48,0.73)}^{\text{‡}}$	0.65(0.48,0.72)	0.60(0.43,0.69)	0.51(0.34,0.67)	0.53(0.40,0.64)	0.33(0.25,0.45)
RF	0.60(0.48,0.65)	0.61(0.55,0.65)	0.59(0.56,0.71)	0.55(0.45,0.64)	0.55(0.47,0.64)	0.31(0.26,0.43)
GRU	${0.66(0.49,0.76)}^{\text{‡}}$	${0.67(0.55,0.71)}^{\text{‡}}$	${0.67(0.58,0.72)}^{\text{‡}}$	${0.62(0.56,0.72)}^{\text{‡}}$	0.51(0.49,0.58)	0.13(0.00,0.32)

BRG, biased random guesser; LR, logistic regression; LDA, linear discriminant analysis; QDA, quadratic discriminant analysis; RF, random forest; GRU, gated recurrent unit neural network.

Best results for each component and metric are specified in bold.

‡ Represents significant improvement with respect to BRG.

### 3.2 Regression

Table 2 shows MAE and Spearman’s (ρ), aggregated across outer folds and 10 random seeds. MAE improvements over a naïve predictor (always predicting the population mean for each subscale) were minimal and not statistically significant. For PF, the GRU model achieves the highest median (p25, p75) Spearman’s ρ at 0.25 (0.11, 0.40), and for CW, ElasticNet reached 0.28 (0.11, 0.38); both results were significantly greater than zero. EE remained the most challenging, with Ridge regression yielding the best result, though not statistically distinguishable from zero.

**Table 2:** Median (p25–p75) Mean Absolute Error (MAE) and Spearman’s ρ for regression prediction of burnout severity across the three SMBM components.

Model	PF		CW		EE	
	MAE	Spearman’s ρ	MAE	Spearman’s ρ	MAE	Spearman’s ρ
Naïve	1.02(0.92,1.17)	–	1.04(0.89,1.14)	–	1.10(1.02,1.20)	–
Lasso	1.02(0.88,1.11)	$0.23(0.05,0.36{)}^{\text{‡}}$	1.05(0.93,1.18)	$0.22(0.11,0.37{)}^{\text{‡}}$	1.12(0.99,1.22)	−0.05(−0.13,0.09)
Ridge	1.05(0.94,1.17)	0.16(−0.04,0.33)	1.06(0.94,1.16)	$0.24(0.04,0.39{)}^{\text{‡}}$	1.09(0.96,1.23)	0.15(−0.03,0.34)
ElasticNet	1.01(0.89,1.11)	$0.24(0.05,0.36{)}^{\text{‡}}$	1.04(0.91,1.18)	${0.28(0.11,0.38)}^{\text{‡}}$	1.12(0.98,1.22)	−0.02(−0.09,0.15)
RF	1.15(0.99,1.31)	−0.18(−0.42,−0.08)	1.06(0.87,1.27)	$0.21(0.03,0.39{)}^{\text{‡}}$	1.18(0.96,1.30)	0.04(−0.09,0.19)
GRU	1.01(0.87,1.21)	${0.25(0.11,0.40)}^{\text{‡}}$	1.11(1.00,1.25)	$0.18(0.00,0.40{)}^{\text{‡}}$	1.12(1.01,1.23)	0.02(−0.24,0.19)

Best results for each component and metric are specified in bold.

‡ Represents significant difference from zero.

### 3.3 Ranking

Table 3 summarizes Spearman’s (ρ) and NDCG across outer folds and 10 random seeds, assessing models’ ability to rank participants by burnout severity. For comparison, we include the average NDCG of a random ranker, computed from 1,000 random rankings per outer fold, and a baseline ranker, i.e., a feed-forward network using only time-invariant features, to assess the added value of wearable-derived data. Notably, the results from the point-wise ranking approach match those obtained from regression, given their equivalence in this context. For the siamese GRU model (S-GRU), we report results using both BCE (S-GRU_*BCE*_) and MRL (S-GRU_*MRL*_) losses. The latter yields the best predictive performance for the PF and CW subscales, with median (p25, p75) Spearman’s ρ values respectively of 0.29 (0.11, 0.46) and 0.25 (0.03, 0.42), and corresponding NDCG scores of 0.93 (0.90, 0.95) and 0.89 (0.85, 0.92), significantly outperforming both the random and baseline rankers. For the EE subscale, the highest performance is achieved by the baseline model using only time-invariant features, though this result is not significantly better than random.

**Table 3:** Median (p25–p75) Spearman’s ρ and Normalized Discounted Cumulative Gain (NDCG) for ranking prediction of burnout risk across the three SMBM components.

Model	PF		CW		EE	
	Spearman’s ρ	NDCG	Spearman’s ρ	NDCG	Spearman’s ρ	NDCG
Random	–	0.89(0.87,0.90)	–	0.85(0.83,0.87)	–	0.84(0.81,0.85)
Baseline	0.00(−0.17,0.14)	0.89(0.87,0.90)	0.12(−0.13,0.32)	0.85(0.82,0.89)	0.08(−0.11,0.24)	0.85(0.80,0.89)
GRU	$0.25(0.11,0.40{)}^{*}$	$0.92(0.89,0.95{)}^{\text{‡},*}$	0.18(0.00,0.40)	$0.88(0.85,0.91{)}^{\text{‡},*}$	0.02(−0.24,0.19)	0.82(0.78,0.86)
S-GRU_*BCE*_	$0.28(0.11,0.44{)}^{*}$	$0.92(0.90,0.96{)}^{\text{‡},*}$	$0.23(0.01,0.40{)}^{*}$	$0.88(0.85,0.91{)}^{\text{‡},*}$	0.02(−0.14,0.21)	0.83(0.79,0.88)
S-GRU_*MRL*_	${0.29(0.11,0.46)}^{*}$	${0.93(0.90,0.95)}^{\text{‡},*}$	${0.25(0.03,0.42)}^{*}$	${0.89(0.85,0.92)}^{\text{‡},*}$	0.03(−0.14,0.21)	0.83(0.81,0.85)

Best results for each component and metric are specified in bold.

‡ Represents significant improvement with respect to the random ranker.

∗ Represents significant improvement with respect to the time-invariant baseline.

## 4 Discussion

When framing the burnout prediction task as binary classification, our findings are broadly consistent with those of our previous work [46], where baseline burnout levels were predicted based on subsequent data, effectively reversing the temporal structure compared to the approach proposed here. We hypothesize that such reversal has limited impact due to burnout’s considerable temporal stability [64]. Relying solely on unobtrusive features, binary classification yields modest predictive performance: PF and CW subscales are captured more accurately than EE, with our best-performing models achieving comparable median ROC AUC for PF (0.66 vs. 0.68), and slight improvements for CW (0.67 vs. 0.61) and EE (0.56 vs. 0.53), though EE performance remains insufficient for practical use, being not significantly better than a random baseline. Moreover, as highlighted also by [65], dichotomizing continuous scores based on predefined thresholds can obscure meaningful variation by discarding the underlying ordinal structure and weakening alignment with psychometrically validated survey instruments. This is avoided when framing burnout prediction as a regression task; however our experiments show that improvements in mean absolute error over a naïve predictor are minimal and not statistically significant. A more nuanced picture emerges when evaluating models using Spearman’s ρ: while absolute predictions remain imprecise, they nonetheless capture meaningful relative structure in the data. This pattern is consistent with recent findings in short-term perceived stress estimation [65], where machine learning regressors failed to outperform baseline models on Symmetric Mean Absolute Percentage Error (SMAPE) but achieved better alignment in terms of rank correlation. Building on these insights, when the goal is to assign scores which reflect individuals’ relative position in terms of burnout risk, rather than to recover exact questionnaire values, explicitly modeling the relative order may be more appropriate. To test this, we reframed burnout prediction as a ranking task and evaluated whether models could capture the ordinal structure of the target scores more effectively. Our results show that the pairwise ranking approach using siamese recurrent networks outperforms the corresponding point-wise sequential model. Specifically, the siamese GRU trained with margin ranking loss (S-GRU_*MRL*_) achieves the best overall performance (PF: ρ=0.29, NDCG = 0.93; CW: ρ=0.25, NDCG =0.89), slightly outperforming its variant trained with binary cross-entropy (S-GRU_*BCE*_), and clearly outperforming the baseline ranker that relies solely on demographic and occupational features, highlighting the added value of wearable-derived physiological trajectories. Notably, while pairwise modeling enhances performance in the sequential modeling setting and yields the best results for PF, the highest rank correlation for CW is still achieved by the ElasticNet regression model (ρ=0.28), which relies on aggregated features. Overall, the results obtained correspond to weak-to-moderate effect sizes [66] for ordinal modeling of PF and CW using unobtrusive data, while EE remains largely out of reach across all frameworks. These findings are broadly consistent with recent work on perceived daily stress prediction from wearable devices [65], which achieved at most ρ=0.19/0.25. Furthermore, unobtrusive modeling could be seen as a lower bound, with room for improvement through the integration of additional data sources.

We acknowledge several limitations in our study and outline directions for improvement. Although the sample size was adequate for exploratory modeling, it constrains both the generalizability of the findings and the predictive performance, especially for deep learning models, which typically benefit from larger datasets. Moreover, the participant cohort did not include individuals clinically diagnosed with burnout; applying the proposed methods to clinically characterized populations, including individuals currently experiencing burnout, those in recovery, and healthy controls, could yield more robust insights. Furthermore, the duration of the observation period may be suboptimal for capturing the gradual onset and progression of burnout [18, 64]. In this context, sustained adherence to the study protocol is especially critical, as missing data—and the imputation procedures required to address it—may introduce subtle biases. This concern is particularly relevant for sequential modeling, where patterns of missingness may themselves correlate with adverse underlying behavioral or physiological states. Regarding feature representation, several unobtrusive yet informative variables previously associated with chronic stress and burnout were unavailable for modeling. These include HRV metrics [33], contextual mobile sensing data (e.g., location entropy, home-work transitions, social proximity) [67], and time-invariant characteristics such as personality traits [68]. Finally, while some burnout sub-dimensions such as PF and CW appear amenable to unobtrusive prediction, others, like EE, are harder to infer passively, potentially requiring self-reported information about life events, either collected via validated surveys [43, 44] or inferred through conversational agents or other LLM-based technologies [69]. We also highlight that, while wearable-based burnout prediction systems can provide valuable organizational and individual insights, their deployment in workplace contexts necessitates robust privacy safeguards, transparent data handling practices, and strong ethical oversight to maintain employee confidence and protect worker rights [70, 71].

Regarding future potential iterations, the current pairwise-ranking siamese recurrent model, akin to the original RankNet formulation [51], treats all pairs of instances with equal importance, regardless of their position in the target ranking. While this uniform weighting can be appropriate for learning globally consistent burnout risk scores, it may be suboptimal when prioritizing individuals at highest risk. This suggests exploring ranking methods that optimize more effectively position-sensitive metrics (e.g., NDCG), for example by adapting metric-aware λ-gradient formulations to recurrent models [51, 72, 73].

## Data availability statement

The datasets presented in this article are not readily available but Research data collected, data processing, and machine learning pipeline for this study can be made available upon reasonable request to M.G. Request for data will be evaluated and responded to in a manner consistent with the study protocol. Requests to access the datasets should be directed to Max Grossenbacher, max@resilient.ai.

## Ethics statement

The studies involving humans were approved by protocol number 628-2023-SPER-AUSLBO, Comitato Etico Area Vasta Emilia Centro, Italy; Clarification of Responsibility Req-2023-00377, SwissEthics, Switzerland. The studies were conducted in accordance with the local legislation and institutional requirements. The participants provided their written informed consent to participate in this study.
