# Supplementary material for: Climbing the ladder: a ranking approach to burnout prediction
Source: Front Digit Health. 2026 Jan 2;7:1694666. doi: 10.3389/fdgth.2025.1694666 (PMC12808414; doi:10.3389/fdgth.2025.1694666)
Supplement: Supplementary file 1 [file Presentation1.pdf]

Supplementary Material

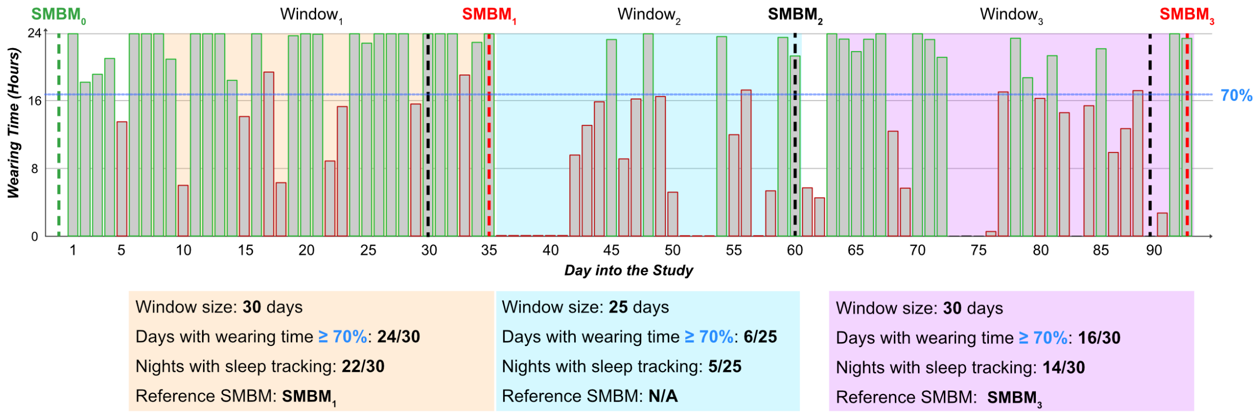

Figure S1. Extraction of SMBM-linked time windows. Each bar represents a day in the study, where the bar height indicates the total wearing time for that day and the bar's outline color reflects the availability of sleep tracking data for the corresponding night. For the sake of clarity, only the first three months are shown. Days in which notifications to fill in the SMBM questionnaire are shown with a dashed black bar, while days in which the SMBM questionnaire was filled in are shown with a dashed red bar. The baseline SMBM is indicated as $SMBM_0$.

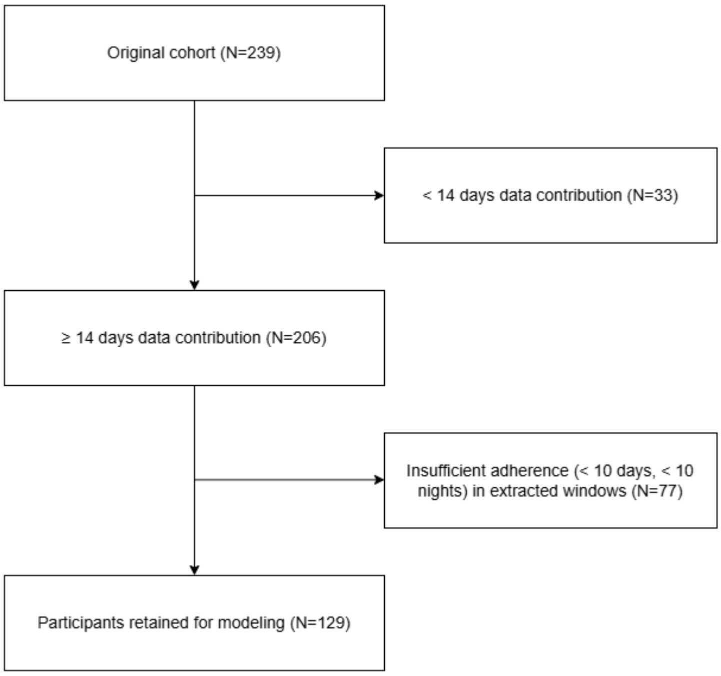

Figure S2. Flowchart of participant filtering for the modeling based on the adherence to the study protocol.

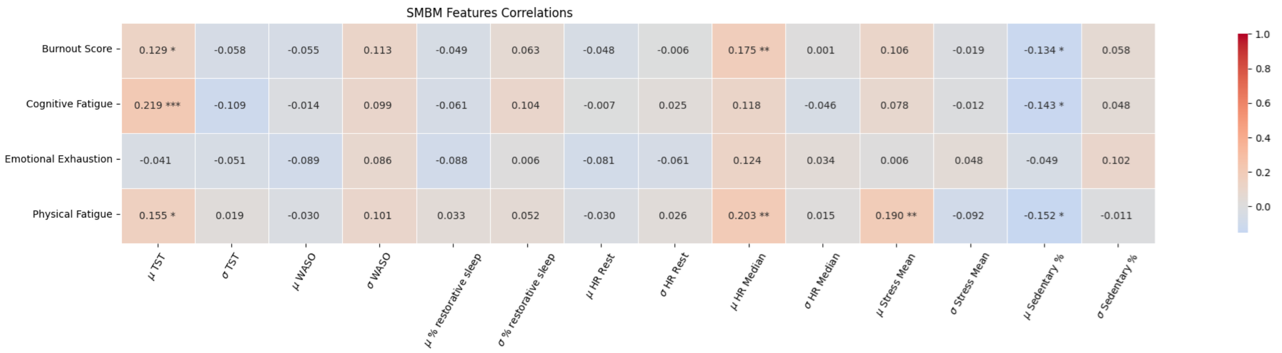

Figure S3. Partial correlation between SMBM scores and mean (μ) and standard deviation (σ) of the chosen features, controlling for all others, across in-study windows.

Table S1. Intraclass Correlation Coefficients (ICCs) and descriptions for SMBM scores and physiological features.

**Variable**	**ICC**	**Description**
SMBM overall score	0.713	Composite burnout score
SMBM CW	0.660	Cognitive weariness subscale
SMBM EE	0.650	Emotional exhaustion subscale
SMBM PF	0.702	Physical fatigue subscale
Total Sleep Time	0.262	Total duration of the sleep session
Wake After Sleep Onset	0.148	Time awake after initially falling asleep
% Restorative Sleep	0.198	Percentage of deep and REM sleep combined
Rest Heart Rate	0.722	Lowest 30-minute moving average heart rate during sleep
Median Awake Heart Rate	0.635	Median heart rate during waking hours
Mean Sleep Stress	0.276	Average overnight Garmin stress score
Sedentary %	0.317	Percentage of time spent sedentary during waking hours

```
Input: In-study windows data {x_i}_{i=1}^N with sequences x_{i,1:T_i} and invariant features x_{i,inv}
Burnout component score {s_i}
Loss type L ∈ {BCE, MRL}
Optimizer Opt
Output: Learned ranking model f_θ(·)

Define scoring function f_θ(x) using a GRU-based encoder followed by fully connected layers.

Generate Training Pairs:
P ← ∅
for i = 1 to N do
    for j = i + 1 to N do
        if |s_i − s_j| ≥ 0.5 then
            Add pair (i, j) to P
        end
    end
end

Pairwise Training Procedure:
foreach pair (i, j) in P do
    Compute scores: ŷ_i ← f_θ(x_i), ŷ_j ← f_θ(x_j)          // Shared weights
    // Loss-specific label definition and loss computation
    if L = BCE then
        // Define ground-truth label in {0,1}
        if s_i > s_j then
            y_ij ← 1
        else
            y_ij ← 0
        end
        ℓ_ij ← BCE(σ(ŷ_i − ŷ_j), y_ij)
    end
    else if L = MRL then
        // Define ground-truth label in {-1, +1}
        if s_i > s_j then
            y_ij ← 1
        else
            y_ij ← −1
        end
        ℓ ← max(0, −y_ij · (ŷ_i − ŷ_j) + ε)
    end
    Compute gradient across all pairs in a batch g ← ∇_θ ℓ
    Update parameters: θ ← Opt(θ, g)
end

return f_θ(·)
```

Algorithm 1: Training procedure for the siamese GRU approach using pairwise ranking losses.

Table S2. Hyperparameter space explored within the scope of the optimization of the methods explored.

Model	Hyperparameter	Values
Logistic Regression	penalty<br>C<br>max_iter<br>tol	[l1, l2]<br>$[0.001, 0.01, 0.1, 1, 10]$<br>$[200, 1000]$<br>$[0.001, 0.01]$
Linear Discriminant Analysis	shrinkage	[auto, $0.1, 0.5, 0.9$]
Quadratic Discriminant Analysis	reg_param	$[0.0, 0.1, 0.5, 1.0]$
Random Forest Classifier	n_estimators<br>max_depth<br>min_samples_split<br>criterion	$[50, 200, 500]$<br>$[2, 5, 12]$<br>$[2, 5]$<br>[entropy, gini]
Lasso Regressor	alpha	$[0.01, 0.1, 0.3, 1.0, 10.0]$
Ridge Regressor	alpha	$[0.1, 0.3, 1.0, 3.0, 10.0]$
ElasticNet	alpha<br>l1_ratio	$[0.01, 0.1, 1.0, 10]$<br>$[0.1, 0.5, 0.7, 0.9]$
Random Forest Regressor	n_estimators<br>max_depth<br>min_samples_split<br>criterion	$[50, 200, 500]$<br>$[2, 5, 12]$<br>$[2, 5]$<br>[mse, absolute_error]
GRU (all modeling paradigms)	optimizer<br>η<br>MRL margin<br>hidden size rnn<br>hidden size ffn<br>dropout<br>batch size<br>epochs<br>patience	Adam<br>$[10^{-3}, 10^{-4}, 10^{-5}, 10^{-6}]$<br>$[0, 0.2, 0.5, 1]$<br>$[8, 16, 32, 64]$<br>$[8, 16, 32, 64]$<br>$[0.0, 0.1, 0.25, 0.4]$<br>$[8, 16, 32]$<br>100<br>10
